# Influence of temperature on the electrochemical window of boron doped diamond: a comparison of commercially available electrodes

**DOI:** 10.1038/s41598-020-72910-x

**Published:** 2020-09-24

**Authors:** Maeve H. S. McLaughlin, Emma Corcoran, Alexander C. Pakpour-Tabrizi, Débora Campos de Faria, Richard B. Jackman

**Affiliations:** 1 London Centre for Nanotechnology and Department of Electronic and Electrical Engineering, University College London (UCL), 17-19 Gordon Street, London, WC1H 0AH UK; 2 Schlumberger Cambridge Research Ltd. (SCR), Madingley Road, Cambridge, CB3 0EL UK

**Keywords:** Engineering, Materials science, Nanoscience and technology

## Abstract

This work compares the electrochemical windows of polished and unpolished boron doped diamond (BDD) electrodes with hydrogen and oxygen terminations at a series of temperatures up to 125 °C. The experiment was run at 5 bar pressure to avoid complications due to bubble formation. An alternative method for determining the electrochemical window is compared to the most commonly used method, which defines the window at an arbitrary current density cut-off (J_cut-off_) value. This arbitrary method is heavily influenced by the mass transport of the electrolyte and cannot be used to compare electrodes across literature where different J_cut-off_ values have been used. A linear fit method is described which is less affected by the experimental conditions in a given measurement system. This enables a more accurate comparison of the relative electrochemical window from various diamond electrode types from reported results. Through comparison of polished and unpolished BDD electrodes, with hydrogen and oxygen surface terminations, it is determined that the electrochemical window of BDD electrodes narrows as temperature increases; activation energies are reported.

## Introduction

Boron doped diamond (BDD) can act as an exceptional electrode material, with the widest electrochemical window of any known material—that is the potential range that can be applied across a working electrode before the onset of either oxidation or reduction of the electrolyte at its surface^1,2^. To date, there has not been a systematic study reported on how the electrochemical window of BDD is affected by temperature. This has particular relevance to the use of BDD electrodes that are employed in extreme environments, such as oil wells, where the electrode can be exposed to temperatures exceeding 150 °C^3^. This work aims to give an initial insight into how temperature impacts the electrochemical window of BDD electrodes.

Experimentally, the electrochemical window of an electrode is determined by measuring a polarisation curve over a potential range wide enough to observe the anodic and cathodic decompositions of the electrolyte at the working electrode; the electrochemical window is the region between these two points^4^. There are several methods that can be employed to define the electrochemical window of an electrode from experimental data. The most commonly used method is to plot the current density (J) in mA/cm^2^ against the applied potential, then to read off the potential at a defined current density cut off (J_cut-off_). The arbitrary choice of the J_cut-off_ value, reported in the range 0.01–5.0 mA/cm^2^, can result in electrochemical windows for the same electrode being quoted to differ by as much as 0.9 V^4–7^. This method is heavily influenced by mass transport of the electrolyte, meaning that changing the concentration of the electrolyte will affect the electrochemical window recorded^4^. Therefore, it is not possible to accurately compare electrochemical windows that have been determined using different J_cut-off_ values. An alternative approach introduced by Olson and Bühlmann has been considered, which was designed to address the inconsistencies that occur when defining the electrochemical window of an electrode by using J_cut-off_ values^8^. In this method, linear fits are made of the three sections of the CV curve, before and after the oxidation and reduction of the electrolyte at the working electrode. The intersections of the linear fits are taken to define the electrochemical window^4,8^. The benefit of this technique is that the defined electrochemical window is less sensitive to the concentration of the electrolyte than when the J_cut-off_ method is used. Also, this method more closely resembles the method of defining the limits of detection of ion-selective electrodes recommended by IUPAC^8,9^. Here, these methods are compared and contrasted with the aim of setting a standard approach for the determination of electrochemical windows from experimental data. To facilitate investigation of the methods described and to define an electrochemical window from experimental results, heavily boron doped diamond electrodes ([B] > 10^20^ atoms/cm^3^) have been used here with two roughnesses: R_A_ ~ 50 nm and R_A_ ~ 50 µm, half of which are hydrogen terminated (BDDH) and half of which are oxygen terminated (BDDO). BDDH has been shown to have a slightly narrower electrochemical window than BDDO, however, there are some sensing applications for which BDDH is more suitable^10,11^. The CV measurements are repeated with each electrode over the temperature range 21–125 °C, to identify how the electrochemical window of BDD is affected by temperature. Although pH will also influence the electrochemical window this is outside the scope of this work. Through use of a buffer system, pH 7, we have fixed the pH throughout the experiments.

## Experimental methods

Electrochemical grade BDD ([B] > 10^20^ atoms/cm^3^) substrates (10 × 10 × 0.5 mm) were purchased from Element Six Ltd. (e6cvd.com). Half of the substrates were unpolished polycrystalline BDD, with surface roughness R_A_ ~ 50 µm. The remaining substrates were polished polycrystalline BDD (pBDD), with surface roughness R_A_ ~ 50 nm. The substrates were laser cut into 3 mm diameter pieces at Laser Micromachining Ltd. (lasermicromachining.com). All chemicals, unless otherwise stated, were purchased from Sigma-Aldrich. Milli-pure water, resistivity 18 MΩ-cm was used throughout (0.22 µm membrane filter).

### BDD surface preparation

Prior to processing, the 3 mm diameter BDD and pBDD substrates were cleaned with a highly oxidising acid to remove adventitious carbon, hydrocarbons, and graphitic carbon on the surface of the diamonds. During the acid clean, the substrates were heated to 200 °C for 10 min in a cleaning solution [ammonium persulfate (20 g) and concentrated sulfuric acid (20 g)] before being placed in the rinsing solution [ammonium hydroxide (10 ml) and hydrogen peroxide (10 ml)] for 10 min^12^. The substrates were rinsed thoroughly with water and dried under N_2_ gas.

The graphitic carbon content at the surface of the electrodes was assessed with a Renishaw micro-Raman spectrometer (532 nm laser source). The microscope was calibrated using a silicon substrate and the Raman analysis was performed with 20 × magnification, 10 s exposure and an average was taken over ten accumulations. WiRE (v 2.0) software was used for data acquisition.

One each of the BDD and pBDD substrates were hydrogen terminated in an AX5010 Seki Technotron Inc. reactor with H-plasma for 10 min, at 400 °C platen temperature (Williamson Dual Wavelength pyrometer), 700 W power, 35 Torr pressure. One BDD and one pBDD substrate were oxygen terminated via ozone treatment in an Ozone Cleaner NL-UV253, under 10^–6^ mbar vacuum, at ozone generation of 10 g/h for one hour. The extent of the hydrogen and oxygen terminations at the substrate surfaces was assessed with contact angle measurements, conducted with a Kruss DSA1 contact angle goniometer, using 4 µl water droplets. Kruss DSA1 v1.80 drop shape analysis software was used to determine the contact angle at the three-phase contact point between the water droplet and the electrode surfaces.

### Electrode preparations

The 3 mm diameter BDD pieces were metallised with Ti–Pt–Au and soldered to Be–Cu pins to form the working electrode. The electrode construction process involved temperatures sufficient for the formation of the required carbide within the diamond-ohmic contact stack given the duration of the process. The bulkhead into which the electrode was placed was machined from PEEK (polyether ether ketone) in-house. A steel counter electrode (4 mm diameter) and a silver wire reference electrode (1.5 mm diameter) were used. The three electrodes were sealed into the bulkhead body using Loctite Hysol 9483 epoxy (rated to 150 °C), which was injected into the channels from behind the pins while applying constant pressure to the front face to avoid leakage of the epoxy to the BDD surface (which would require vigorous polishing to remove, thus destroying the terminations). The bulkhead did require some polishing before the electrochemical measurements to remove dust and other residues left on the electrode surfaces following the sealing of the bulkhead with epoxy. Contact angle measurements were made after the electrodes were polished with 3 µm diamond slurry (Kemet International Ltd.) on a PSU-M polishing pad (Kemet International Ltd.) to assess the extent of the hydrogen and oxygen terminations following this polishing procedure. These measurements were made with a Kruss DSA1 contact angle goniometer, using 4 µl water droplets, and Kruss DSA1 v1.80 drop shape analysis software was used to determine the contact angle at the three-phase contact point between the water droplet and the electrode surfaces.

### Electrochemical measurements

Cyclic voltammetry (CV) measurements, were used to determine the electrochemical window of each BDD working electrode in a 1 M phosphate buffer electrolyte (0.025 M K_2_HPO_4_, 0.025 M KH_2_PO_4_, 0.1 M KCl) doped with 0.5 mM 3-ferrocenophane sulfonate (prepared in-house at SCR) as a pseudo reference system. Before the CV measurements, the electrodes were polished with a 3 µm diamond slurry (Kemet International Ltd.) on a PSU-M polishing pad (Kemet International Ltd.) and rinsed thoroughly with water.

The bulkhead was placed in a PEEK flow cell using Viton o-rings to seal it into place, then the cell was placed in a steel box (to aid heat transfer). A thermocouple was inserted into the bottom of the cell to measure the temperature. The cell was connected to a two-channel syringe pump system (Syrris Asia pump) via Hastelloy fittings inside an oven. The flow line to the electrode was held at 5 bar pressure, to prevent the electrolyte from boiling at the elevated temperatures measured. The solution was injected into the cell, via a heating coil inside the oven, to fill the sensing chamber above the electrode (approx. 1 mm high). Measurements were made after the temperature in the cell was stable for 10 min. The flow was diverted to waste just before measurement, so that static conditions were achieved at the electrode surface whilst taking a scan and reopened after measurement to flush the sensing chamber. CV staircase scans with upper vertex potential 1.5 V, lower vertex potential − 1.9 V and 0.0085 V step potential were used to determine the electrochemical windows. The CV scans were repeated at the scan rates 01, 0.5, and 1 V/s for each temperature measured (21, 50, 75, 100, and 125 °C) with each electrode. As the electrochemical window is independent of the scan rate, the data from each scan rate was combined with the repeats made using each electrode to determine the error in the observed current.

### Electrochemical window determination

**Figure 1:**
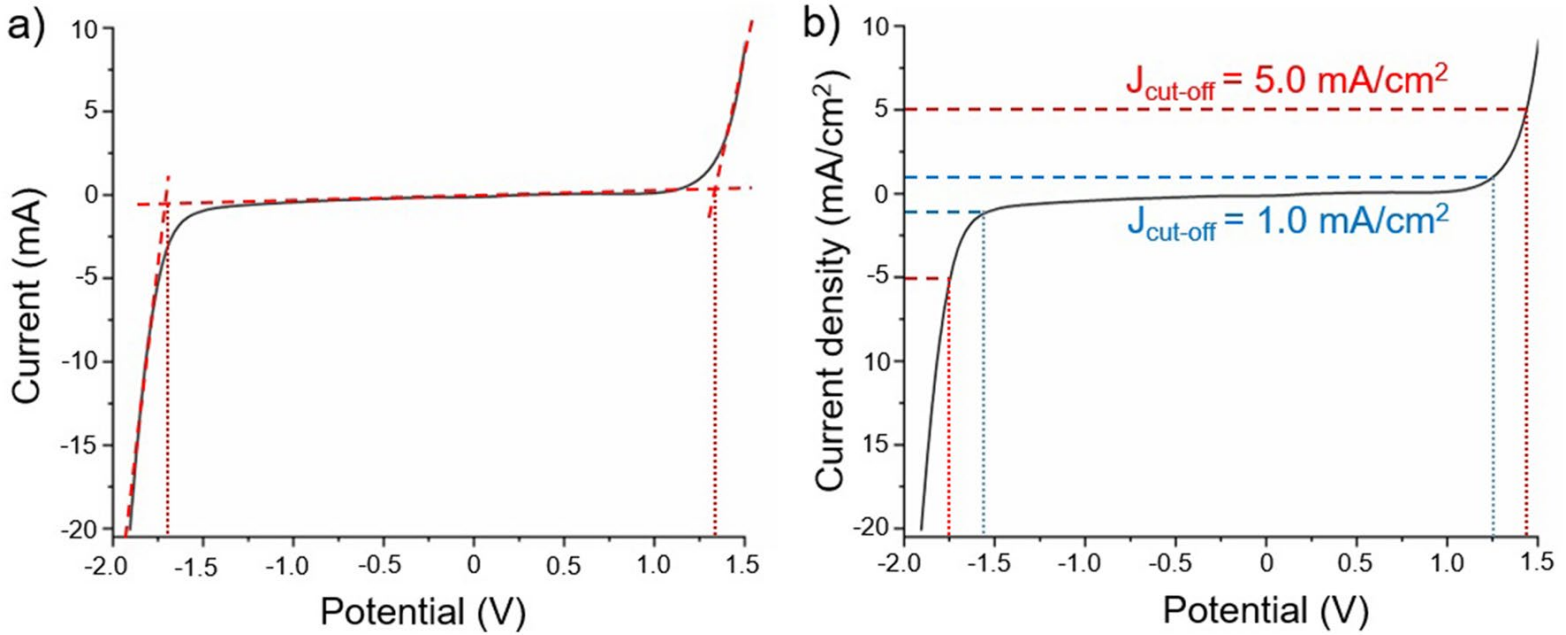
Comparison of the two methods identified for determining the electrochemical window of an electrode from experimental data (BDD working electrode in a 1 M phosphate buffer electrolyte (0.025 M K_2_HPO_4_, 0.025 M KH_2_PO_4_, 0.1 M KCl) doped with 0.5 mM 3-ferrocenophane sulfonate as a pseudo reference system). The methods are: (**a**) taking the intersection of the linear fits of the CV curve (the crossing points of the dashed lines)^4,9^ and (**b**) the well-established J_cut-off_ method that determines the electrochemical window by a predefined current density (J) value of 1.0 or 5.0 mA/cm^2^.

Two methods for determining the electrochemical window from the experimental data have been explored, as illustrated in Fig. 1, in which the vertical dotted lines represent where the electrochemical window is defined. Figure 1a shows the current/potential curve obtained experimentally, to which three linear fits have been applied. The electrochemical window is defined as the potential window between the two intersections of the linear fits. Figure 1b illustrates the J_cut-off_ method for the J_cut-off_ values 1.0 mA/cm^2^ and 5.0 mA/cm^2^. Here the electrochemical window is defined as the potential window between the points where the J_cut-off_ value intersects with the current density/potential curve.

## Results

### BDD characterisation

**Figure 2:**
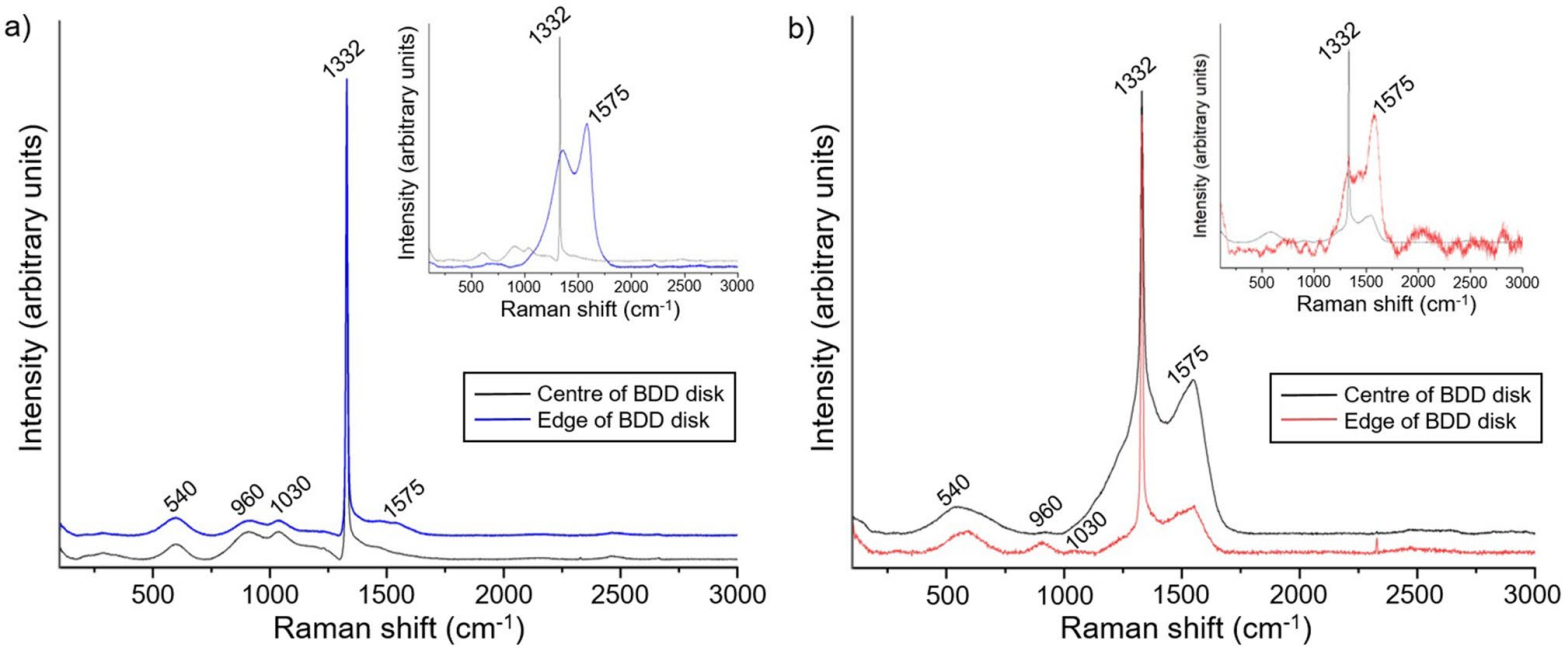
(**a**) Raman spectrum of unpolished BDD substrate revealing the distinctive 1332 cm^−1^ peak of the diamond carbon phase, and (inset) Raman spectrum before the substrate was acid cleaned and (**b**) similar spectrum for the polished pBDD substrate materials.

The proportion of non-diamond carbon and sp^3^ diamond carbon on the BDD surfaces was assessed with Raman spectroscopy before and after the acid cleaning process described in “BDD surface preparation” section (Fig. 2).

The extent of the hydrogen and oxygen terminations imparted by the processes described in “BDD surface preparation” section were qualitatively assessed with contact angle measurements. The measurements were repeated after the electrodes were polished with 3 µm diamond slurry. These give an insight into the hydrophilic/hydrophobic nature of the substrate surfaces before and after they were polished (Table 1).

**Table 1:** Three-phase contact angle on each substrate surface from contact angle measurements with 4 μml water droplets.

Electrode	After substrate termination	After polishing with 3 µm diamond slurry
	Contact angle (°)	Contact angle (°)
Unpolished BDDH	92 ± 1.0	78 ± 1.0
Polished BDDH	98 ± 0.5	55 ± 1.5
Unpolished BDDO	55 ± 1.0	31 ± 1.0
Polished BDDO	36 ± 0.5	29 ± 1.0

### Electrochemical measurements

**Figure 3:**
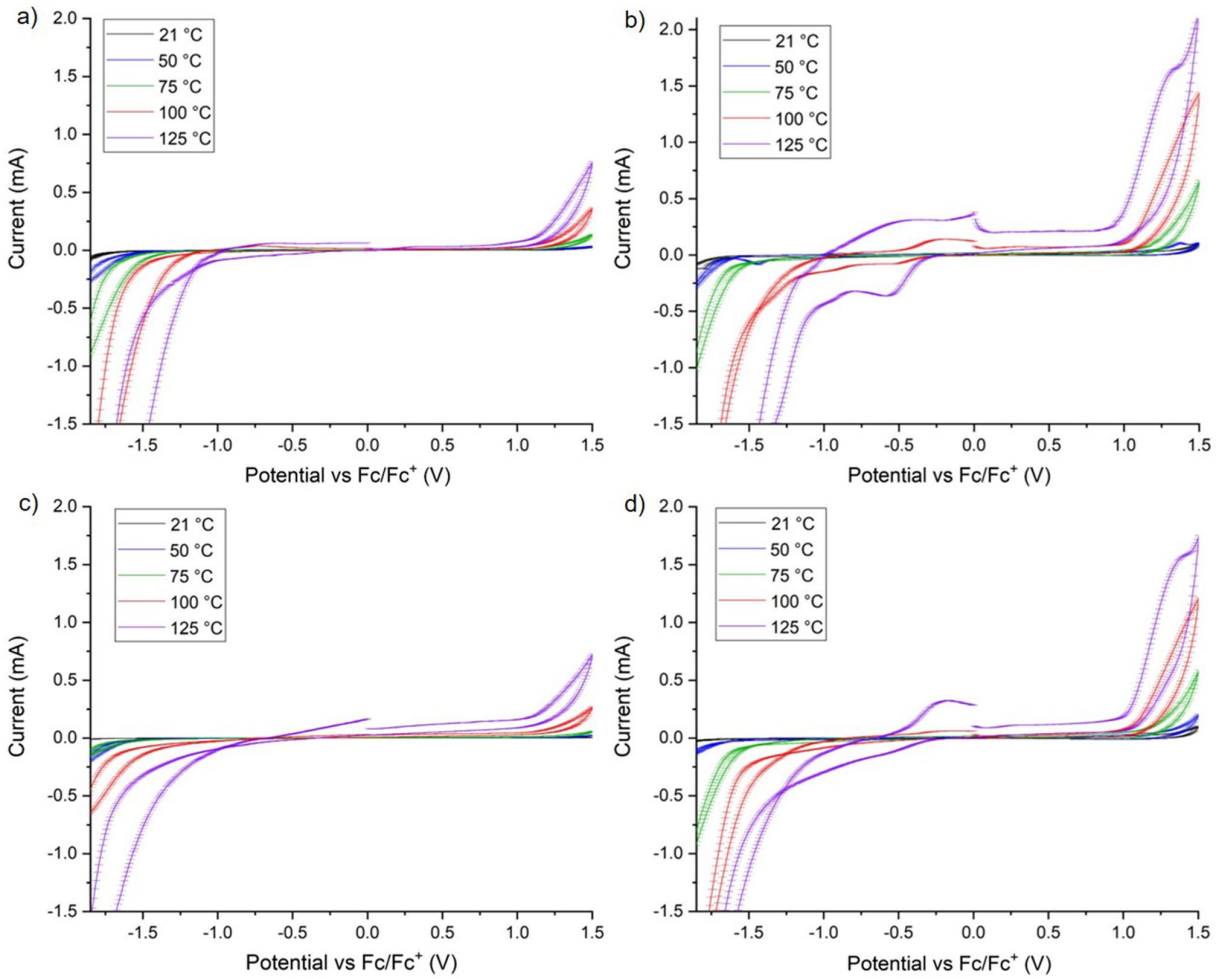
Combined spectra of the CV average scans and standard deviation across the full temperature range measured for each electrode (**a**) unpolished BDDH, (**b**) polished BDDH, (**c**) unpolished BDDO and (**d**) polished BDDO at a scan rate of 0.5 V/s (similar results obtained at scan rates 0.1 V/s and 1.0 V/s are in the Supplementary Information).

The CV measurements made with each electrode were repeated four times at each temperature measured (21, 50, 75, 100, and 125 °C). The average observed current from these repeats with the standard deviation for these values is plotted in Fig. 3.

**Figure 4:**
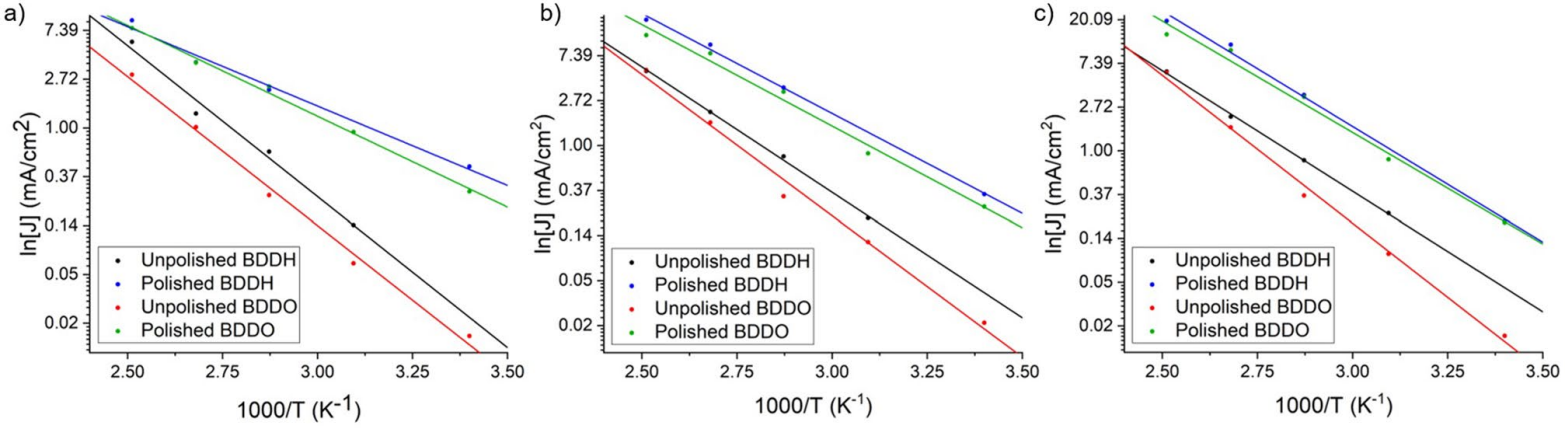
Arrhenius plots of ln[J] versus inverse temperature at the potential − 1.4 V, which is part of the way through the electrolyte oxidation at all temperatures from 21 to 125 °C for each electrode, for each scan rate: (**a**) 0.1 V/s, (**b**) 0.5 V/s and (**c**) 1.0 V/s.

The activation energy for the oxidation potential for each electrode (Table 2) was determined from the slopes of Arrhenius plots of ln(current density) against the inverse temperature in Kelvin at the applied potential − 1.4 V (Fig. 4), which is part way through the oxidative curve for the electrodes at each temperature measured. By plotting the current densities as a function of inverse temperature we can determine the activation energies of the hydrogen evolution reaction with the Arrhenius equation (Eq. 1) where J is the current density, A is the Arrhenius pre-exponential factor, E_A_ is the activation energy, k_b_ is the Boltzmann constant and T is the absolute temperature.

1 $$J = A\exp \left( {\frac{{ - E_{A} }}{{k_{b} \cdot T}}} \right)$$

**Table 2:** Activation energies and standard error for each electrode derived from the Arrhenius plots in Fig. 4.

Electrode	Activation energy (eV)	Error (eV)
Unpolished BDDH	6.68 × 10^7^	± 0.25 × 10^7^
Polished BDDH	5.02 × 10^7^	± 0.68 × 10^7^
Unpolished BDDO	7.41 × 10^7^	± 0.23 × 10^7^
Polished BDDO	5.16 × 10^7^	± 0.46 × 10^7^

### Electrochemical window determination

**Figure 5:**
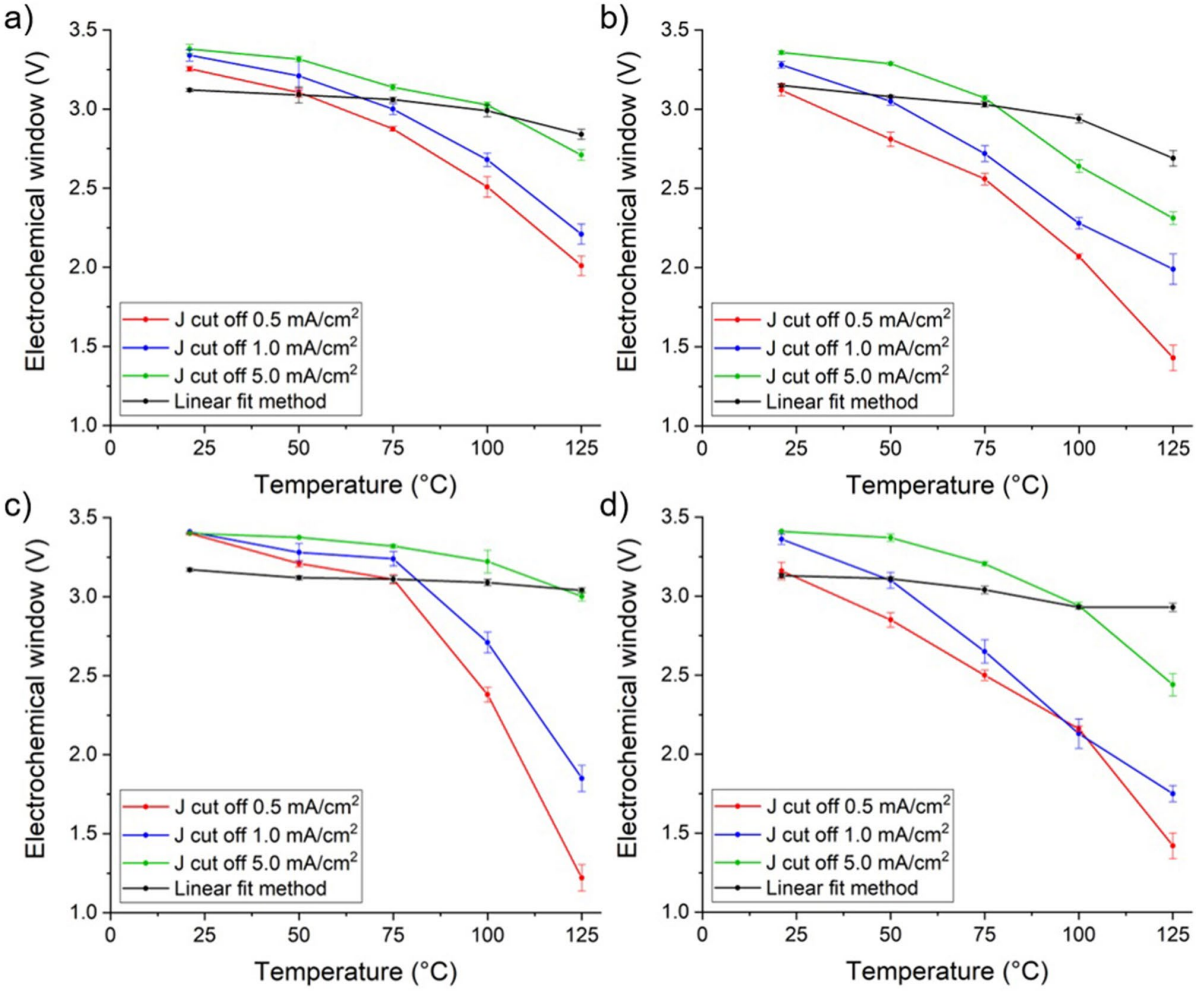
Comparison across the temperature range 21–125 °C of the electrochemical window of each electrode (**a**) unpolished BDDH, (**b**) polished BDDH, (**c**) unpolished BDDO and (**d**) polished BDDO, as determined by the J cut off method at 0.5 mA/cm^2^, 1.0 mA/cm^2^, 5.0 mA/cm^2^ and the intersection of linear fits method described in “Electrochemical window determination” section.

The electrochemical window of each electrode was determined by the two methods described in “Electrochemical window determination” section. A comparison of how the electrochemical windows of the electrodes were affected by temperature with each of these methods is shown in Fig. 5. The electrochemical windows determined with the J_cut-off_ method are heavily influenced by the choice of J_cut-off_ value. In Fig. 5 this is demonstrated by comparison of the electrochemical windows determined for each electrode with the linear fit method and the J_cut-off_ method using the J_cut-off_ values 0.5, 1.0, and 5.0 mA/cm^2^.

## Discussion

The proportion of non-diamond carbon and sp^3^ diamond carbon at the BDD surfaces before and after the acid cleaning process was analysed with Raman spectroscopy (Fig. 2). Before the substrates were acid cleaned (inset Fig. 2) the characteristic 1332 cm^−1^ diamond carbon peak is clearly seen in the centre of the BDD disks. However, at the edge of the disks the 1332 cm^−1^ peak is broad and has a lower intensity than the non-diamond carbon G peak at 1575 cm^−1^. This indicates that when the BDD disks were laser-cut from the 10 × 10 cm^2^ squares purchased from Element Six Ltd. non-diamond carbon was most likely sputtered onto the edge of the diamond surface as a result of the laser ablation^12^. The acid cleaning procedure removed a significant proportion of this non-diamond carbon from the BDD surfaces, as indicated by the presence of the characteristic 1332 cm^−1^ diamond carbon peak at both the centre and edge of each substrate, at a significantly greater intensity than the 1575 cm^−1^ peak. The shape of the 1332 cm^−1^ peak after the acid clean is indicative of the crystalline quality of the BDD substrates, as a lower quality diamond that contains more defects would have a shorter phonon lifetime and broader line width than seen in Fig. 2^1^.

Comparison of the 1332 cm^−1^ and 1575 cm^−1^ peaks can be used to assess the relative proportions of diamond and non-diamond carbon at the substrate surfaces. Although, as the relative intensities of the two peaks are dependent on the grain size, film stress, doping density, and excitation wavelength used, we can only qualitatively compare the two peaks; in the visible region, the sensitivity to sp^2^ materials is typically 100 times that of sp^3^^13^. Following the acid clean, the proportion of sp^2^ carbon at the centre of the substrates is very low when compared to literature values where Raman spectroscopy is supported by other techniques^14,15^. The intensity of the 1575 cm^−1^ peak remains high in relation to the 1332 cm^−1^ diamond peak at the edge of the polished BDD substrate, even after the acid clean. This is likely due to the polishing process introducing damage to the diamond surface, which has not been completely removed during the acid clean.

The broad peaks between 500 cm^−1^ and 1030 cm^−1^ in the Raman spectra for both the polished and unpolished BDD substrates indicate a high boron doping concentration (> 10^20^ boron atoms/cm^3^) within the diamond structures (Fig. 2)^16^. The Fano resonance (asymmetry at the base of the 1332 cm^−1^ peak) further confirms the high concentration of boron in the diamond lattices, as this relates to the onset of metal-like conductivity in the diamond, which is a result of the boron impurity band transitioning into a continuum state^17^.

The average surface termination across the electrodes was assessed with contact angle measurements (Table 1), after the terminating processes described in “BDD surface preparation” section and again after the electrodes were polished with 3 µm diamond slurry, before the CV measurements. Determination of the exact wetting angle from inspection of contact angle measurements is open to error, however, the values for the contact angle on the polished and unpolished BDDH electrodes after termination are consistent with a strongly hydrophobic and therefore hydrogen terminated surface, when compared to literature^18^. After polishing with 3 µm diamond slurry the contact angles on the unpolished and polished BDDH were reduced to 78° and 55° respectively, which corresponds to the hydrogen termination being damaged and the surfaces therefore becoming more hydrophilic. Both sets of contact angles for the unpolished and polished BDDO electrodes are indicative of a hydrophilic and therefore predominantly oxygen terminated surface^19^. The contact angle for a fully oxidised diamond surface would be expected to be < 30°, although, diamond is considered to be predominantly oxygen terminated when the contact angle is between 0 and 60^1,20^.

Contact angle measurements are heavily influenced by the environment of the measurement, not just the electrode itself, especially in the case of small electrode sizes. This means that absolute contact angle measurements contain too much error for direct interpretation. However, relative measurements can still be informative. Here, BDDO contact angles were considerably smaller than for BDDH, as expected. The high temperature measurement did not, at least for the duration of the experiments carried out here, lead to any significant change in either. BDDH with a contact angle of 78° ± 1.0° after polishing was subsequently measured at 78° ± 2.0° after the high temperature measurements; BDDO (31° ± 1.0° after polishing) was subsequently measured at 30° ± 1.5°.

As the temperature of the CV measurements was increased from 21 to 125 °C the onset of oxidation and reduction of the electrolyte at the working BDD electrodes occurred at smaller positive and negative potentials. Therefore, the electrochemical window for each electrode narrowed as the temperature was increased (Fig. 3).

The Arrhenius plots in Fig. 4 show that the current densities of the hydrogen evolution reaction (reduction of the electrolyte) recorded at − 1.4 V for each electrode are temperature dependent. The anomalous peaks at − 0.5 V, − 0.2 V, and 1.25 V lead to analogously high current densities for the hydrogen evolution peak at − 1.4 V for both polished electrodes (BDDH and BDDO), so these points were omitted from the data used to make the Arrhenius plots. The activation energies derived from the Arrhenius plots are shown with the standard error in these values in Table 2. The activation energies are higher and with smaller errors for the unpolished electrodes compared to the polished electrodes, which corresponds to the wider electrochemical windows reported for the unpolished electrodes in Fig. 5, for both methods of determination (with J_cut-off_ 0.1 mA/cm^2^).

The unpolished electrodes have a lower proportion of sp^2^ to sp^3^ carbon at their surface (Fig. 2) than the polished electrodes, meaning that a wider electrochemical window is expected^21^. When the proportion of sp^2^ carbon is higher, as with the polished electrodes, the oxidation mechanism at the BDD surfaces is affected, leading to changes in the electrocatalytic properties of the electrode surface and reducing the barrier activation to the evolution of hydrogen and reduction of oxygen at the electrode surface, therefore reducing the electrochemical window^21,22^. The activation energies are slightly higher for the BDDO electrodes than the BDDH electrodes (both polished and unpolished), as reported previously^10^. However, the different terminations resulted in significantly smaller changes in the activation energy and electrochemical windows reported than the different surface roughness of the electrodes.

In Fig. 3b,d there are analogous peaks at − 0.5 V, − 0.2 V, and 1.25 V for the CV spectra at 125 °C for both polished electrodes (BDDO and BDDH). As nothing changed in the system other than BDD electrode used, these peaks must be a result of the changes induced by the higher sp^2^/sp^3^ carbon ratio at the surfaces of the polished electrodes^21,22^. In existing literature there are no reports of CV spectra for similar electrodes above 100 °C, so we are unable to make comparisons between this change in the CV spectra and other results. Further investigation is needed.

**Figure 6:**
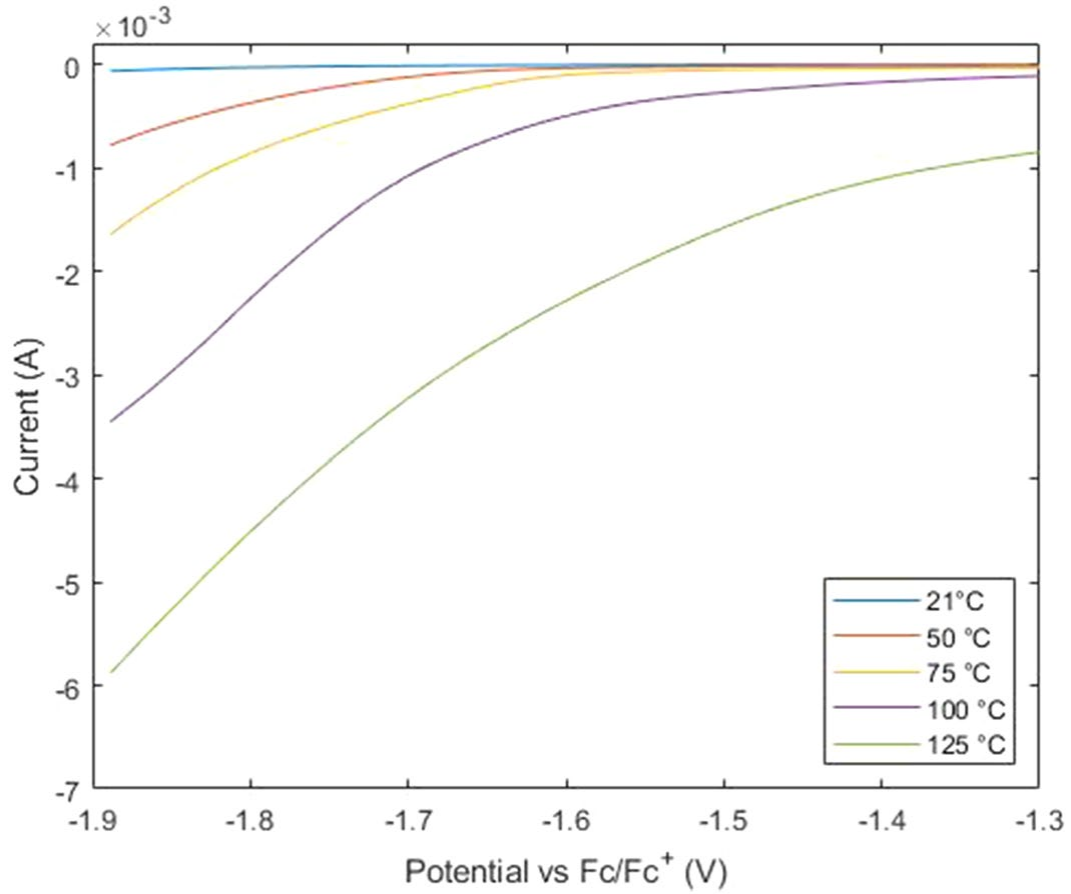
Oxygen reduction reaction at the polished BDDO electrode with increasing temperature from 21 to 125 °C.

The linear fit method resulted in electrochemical windows that were apparently narrower at room temperature, than when the J_cut-off_ method was used, and less impacted by increasing temperature (Fig. 5). As the temperature of the CV measurements increased and the electrochemical windows narrowed, the linear fits of the curve remained similar. The significant change is that the curves at the beginning of oxidation and reduction of the electrolyte become shallower as the temperature increases (Fig. 6). The change in curvature is more subtle to the linear fit method, meaning that for the same experimental results the electrochemical window will appear to undergo less change with increasing temperature than when the J_cut-off_ method is used.

To demonstrate the extent to which the arbitrary choice of the J_cut-off_ value affects the determined electrochemical window, we compared three J_cut-off_ values: 0.5, 1.0, and 5.0 mA/cm^2^ with the linear fit method (Fig. 5). In Fig. 5c the electrochemical window determined with J_cut-off_ 0.5 mA/cm^2^ is 2.31 V, but when determined with J_cut-off_ 5 mA/cm^2^ it is 0.88 V lower, at 1.43 V. This is a significant difference. It is not possible to compare the electrochemical windows quoted across literature where different J_cut-off_ values have been used and as this method is heavily influenced by the mass transport of the electrolyte it is only accurate to compare electrochemical windows for electrodes that have been determined under the same experimental conditions.

Particularly for the unpolished BDDH and BDDO electrodes (Fig. 5a,c), the linear fit method has a similar trend to the J_cut-off_ method using 5.0 mA/cm^2^. This highlights the importance of choosing an appropriate J_cut-off_ value because it is shown here that the linear fit method is less sensitive to changes in the curvature of the hydrogen evolution or oxygen reduction reactions (Fig. 6), so a less accurate method of determining the electrochemical window. This overshadows the aim of the linear fit method, to determine electrochemical windows by a method that is less influenced by the concentration of the electrolyte than the J_cut-off_ method, meaning that comparison between literature is possible^4^.

The electrochemical windows reported in these results were shown to narrow as the temperature of the measurements increased for each of the electrodes. This means that when using these electrodes in high temperature environments the user would need to consider the range of ions that it would be possible to identify, as redox peaks outside the electrochemical window would not be detected. However, each electrode would still be appropriate for use at high temperatures when analysing redox peaks within this range. The unpolished electrodes would offer the most versatility as these electrodes have the widest electrochemical windows, so allow the detection of the widest range of ions.

## Conclusions

This work has been an investigation into how the electrochemical window of BDD electrodes is affected by temperature and how the method used to determine the electrochemical window from experimental data can affect the apparent result. The experiment was run at 5 bar pressure to avoid complications due to bubble formation. For every electrode, the electrochemical windows became narrower as the temperature increased from 21 to 125 °C, which is to be expected since the redox reaction is thermally activated. The widest electrochemical windows were reported for the unpolished electrodes, which have a lower proportion of sp^2^ carbon at their surfaces in comparison to the polished electrodes. The influence on the ratio between sp^2^ and sp^3^ carbon at the electrode surfaces was shown to have a much more significant impact on the electrochemical window value reported than the hydrogen or oxygen termination of the diamonds.

A reliable standard procedure for determining electrochemical windows has been sought that could be used to make accurate comparisons across the published literature, unlike the commonly used J_cut-off_ method. The linear fit method proposed by Olson and Bühlmann is less affected by the assumptions required in the J_cut-off_ method; the application of this method to the results reported across the published literature would therefore enable a more accurate comparison of the variation in the value of the electrochemical window due to varying measurement conditions. This is valuable to select one type of electrode versus another. However, it is the electrochemical window determined when using the J_cut-off_ method, with a carefully selected J_cut-off_ value, that will give the effective electrochemical window that will be relevant for a given experimental arrangement where sensitivity to a given current level is required.

## Supplementary information

Supplementary file1
