# Supplementary material for: Influence of temperature on the electrochemical window of boron doped diamond: a comparison of commercially available electrodes
Source: Sci Rep. 2020 Sep 24;10:15707. doi: 10.1038/s41598-020-72910-x (PMC7518440; doi:10.1038/s41598-020-72910-x)
Supplement: Supplementary file 1 — Supplementary file1 [file 41598_2020_72910_MOESM1_ESM.docx]

Supplementary Information

Influence of temperature on the electrochemical window of boron doped diamond: A comparison of commercially available electrodes

Maeve H. S. McLaughlin^1^, Emma Corcoran^2^, Alexander C. Pakpour-Tabrizi^1^, Débora Campos de Faria^2^, Richard B. Jackman^1^

^1^London Centre for Nanotechnology and Department of Electronic and Electrical Engineering, University College London (UCL), 17-19 Gordon Street, London, WC1H 0AH, UK.

^2^Schlumberger Cambridge Research Ltd. (SCR), Madingley Road, Cambridge, CB3 0EL, UK

^*^Author for correspondence, e-mail: [r.jackman@ucl.ac.uk](mailto:r.jackman@ucl.ac.uk)

**
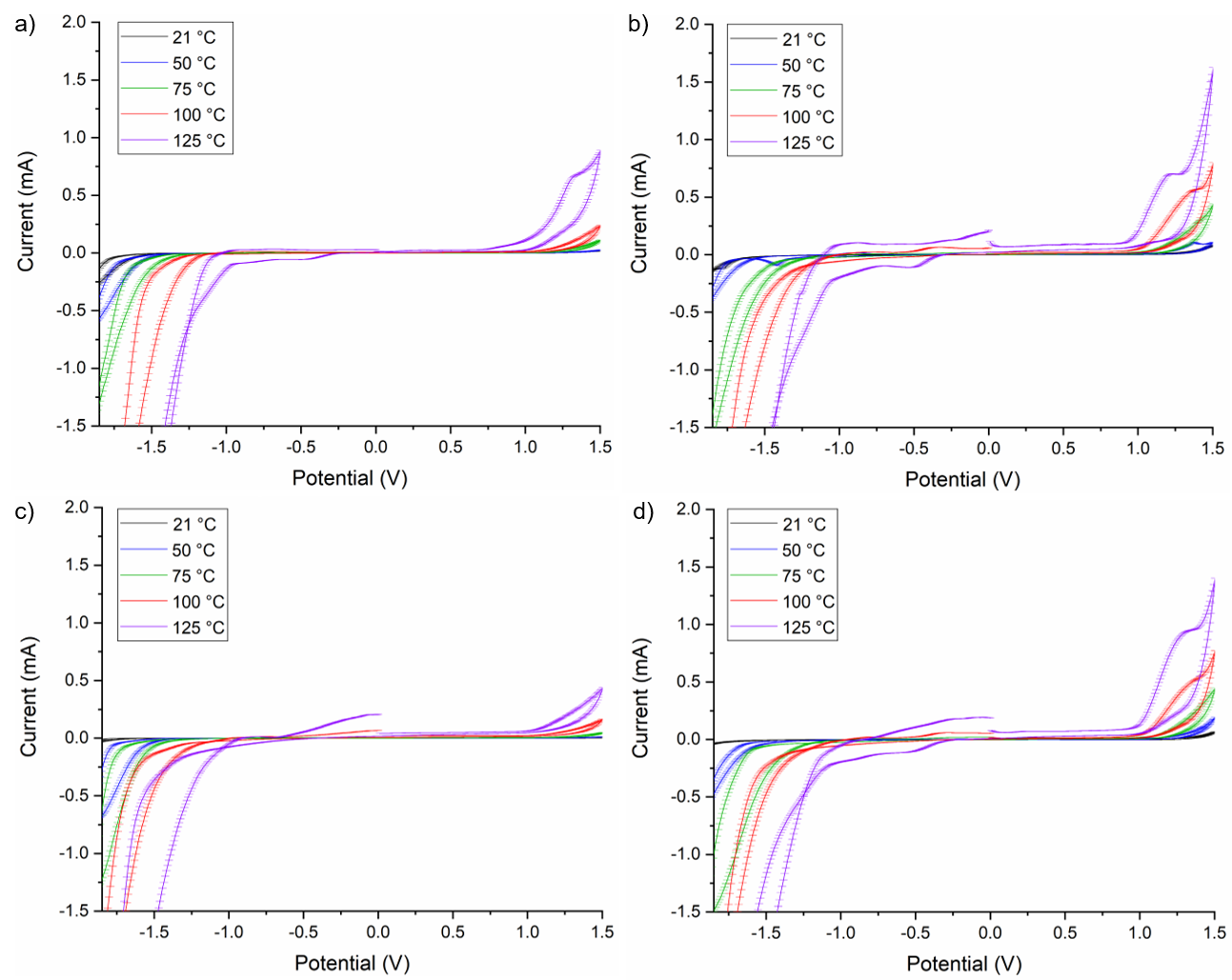
**

Supplementary figure 1. Combined spectra of the CV average scans and standard deviation across the full temperature range measured for each electrode a) unpolished BDDH, b) polished BDDH, c) unpolished BDDO and d) polished BDDO at a scan rate of 0.1 Vs^-1^.


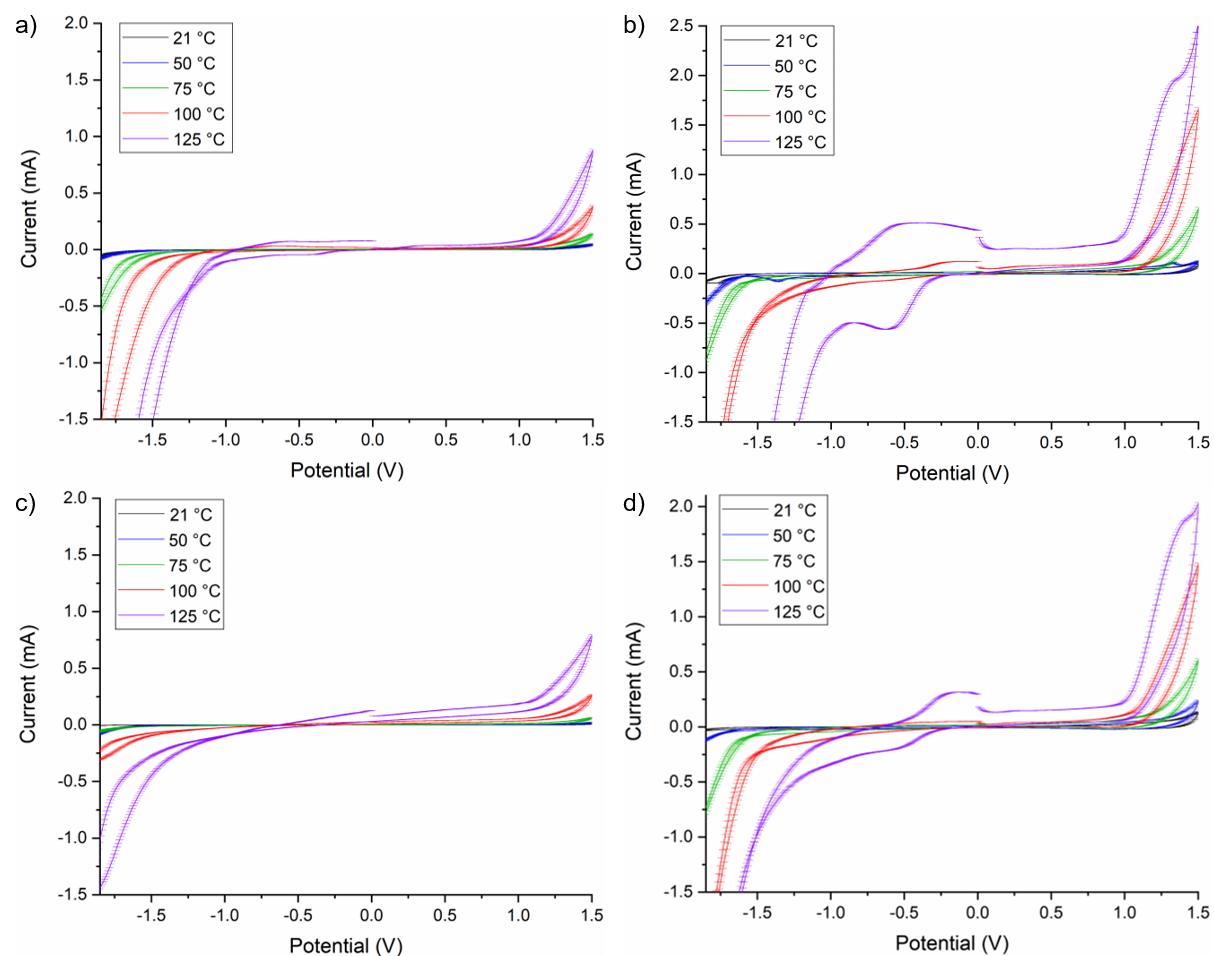


Supplementary figure 2. Combined spectra of the CV average scans and standard deviation across the full temperature range measured for each electrode a) unpolished BDDH, b) polished BDDH, c) unpolished BDDO and d) polished BDDO at a scan rate of 1.0 Vs^-1^.
